# Cerebral hemodynamics and endothelial function in patients with Fabry disease

**DOI:** 10.1186/1471-2377-13-170

**Published:** 2013-11-11

**Authors:** Tomás Segura, Oscar Ayo-Martín, Isabel Gómez-Fernandez, Carolina Andrés, Miguel A Barba, José Vivancos

**Affiliations:** 1 Department of Neurology, Hospital General Universitario de Albacete, C/Hermanos Falcó S/N, Albacete 02006, Spain; 2 Medicine Department, Hospital General Universitario de Albacete, Albacete, Spain; 3 Neurology Department, Hospital Universitario de La Princesa, Madrid, Spain

**Keywords:** Fabry disease, Hemodynamics, Chemokines, Endothelium

## Abstract

**Background:**

Cerebral vasculopathy have been described in Fabry disease, in which altered cerebral blood flow, vascular remodelling or impairment of endothelial function could be involved. Our study aims to evaluate these three possibilities in a group of Fabry patients, and compare it to healthy controls.

**Methods:**

Cerebral hemodynamics, vascular remodelling and systemic endothelial function were investigated in 10 Fabry patients and compared to data from 17 healthy controls. Transcranial Doppler was used to study blood flow velocity of intracranial arteries and cerebral vasomotor reactivity. For the study of vascular remodelling and endothelial function, intima-media thickness of common carotid arteries, flow-mediated dilation in brachial artery and serum levels of soluble VCAM-1, TNF-α, high-sensitive CRP and IL-6 were measured. Differences between groups were evaluated using appropriate tests.

**Results:**

No relevant differences were observed in cerebral hemodynamic parameters, intima-media thickness or flow-mediated dilation. There was a trend for low serum levels of IL-6 and high serum levels of TNF-α and high-sensitive CRP in Fabry patients; plasma concentrations of soluble VCAM-1 were significantly higher in Fabry disease patients than in healthy volunteers (p = 0.02).

**Conclusions:**

In our sample, we did not find relevant alterations of cerebral hemodynamics in Fabry disease patients. Increased levels of plasmatic endothelial biomarkers seem to be the most important feature indicative of possible vascular dysfunction in Fabry disease patients.

## Background

Anderson Fabry disease (FD) (OMIM 301500) is an X-linked inherited sphingolipid storage disorder caused by deficiency of lysosomal enzyme alpha-galactosidase A (GLA). This illness is characterized by progressive accumulation of glycosphingolipids, mainly globotriaosylceramide (Gb3), in lysosomes in a variety of cell types, including renal cells, myocardial cells, heart valve fibrocytes, nervous system cells, and endothelial cells of blood vessels [1-7].

FD is associated with a broad range of clinical symptoms [3], including pain and paresthesias in the extremities, sensorineural hearing loss, angiokeratoma, corneal and lenticular opacities, hypohidrosis, cardiac and renal dysfunction, gastrointestinal symptoms, TIA and stroke [3,5,7]. The disease can be diagnosed measuring alpha-galactosidase A activity in leukocytes or plasma and analyzing genotype (mutation analysis of GLA gene). Once the diagnosis is made, FD can be treated with enzyme replacement therapy (ERT), commercially available since 2001 [3,5,8]. Monitoring of FD progression is based on clinical, radiological, and laboratory assessments [9].

Cerebral micro- and macro-angiopathy are hallmarks of FD [6] and cerebrovascular ischemic events (brain infarctions or transient ischemic attacks) are present in over 25% of patients, and the frequency increases with age. In fact, it is probable that stroke could be the first manifestation of the disease in some patients [10] and one of the most common clinical manifestations in women [11]. An etiology of each single event is hard to identify, and it is likely to be mixed even in the same patient at different times: arteriolar obstruction, arteriosclerosis, arterio-arterial embolism and cardiogenic embolism are all potential mechanisms [8].

Of note, despite its potential severity, there is currently no early marker for cerebrovascular risk that allows optimization of the treatment of patients affected by FD (either hemizygotic males or heterozygotic females), nor is it possible to investigate the value of ERT in reducing that risk. Among possible markers to cerebral impairment in FD, parameters of brain hemodynamics, with controversial results, have been studied [12-15].

In this context, the aim of our study was to investigate the potential presence of alterations in cerebral hemodynamics (using transcranial Doppler to measure brain blood flow velocities and cerebral vasomotor reactivity), as well as the existence or not of vascular remodelling (measuring intima-media thickness and the distance between adventitial layers in common carotid arteries) and the presence of endothelial dysfunction (using the reactive brachial hyperaemia test and levels of endothelium released markers) in patients of both sexes diagnosed of FD in comparison with healthy volunteers.

## Methods

A total of 27 subjects over age 18 years and agreeing to take part in the study were recruited and centrally studied in our neurosonology laboratory. The sample included 17 age and sex-matched healthy volunteers (12 females and 5 males, age range 18–61 years) and 10 patients diagnosed with FD (6 females and 4 males, age range 18–61 years). All patients carried a mutation associated with a classic phenotype of FD. All males had a low or very low GAL enzyme activity in plasma (between 1–15 nmol/h/mg). In women, the diagnosis had been made through the identification of a pathogenic GLA mutation on molecular genetic testing, although in three of the six women enzymatic activity was also low, less than 15 nmol/h/mg. In the Fabry group, all males and two females were receiving ERT. Three males were taking this treatment for 5 years, one since 7 years and one for 3 years. One of the females took the treatment for 5 years and the other female since one year before the study measurements.

All patients with FD were selected from the Spanish Fabry Outcome Survey (FOS) database. The work was part of a wider project, within an educational grant awarded to the Department of Neurology of Albacete General Hospital. The study complied with the ethical principles of the Spanish Fabry Outcome Survey Project protocol and was approved by the Albacete General Hospital Research Ethics Committee. All patients as well as control subjects provided informed consent.

Exclusion criteria included the existence of an unstable cardiovascular disorder, previous cerebrovascular symptoms, anemia or severe obstructive pulmonary disease and the absence of a transcranial sonographic window.

For the study of brain hemodynamics, both determination of blood flow in brain arteries and measurement of cerebral vasomotor reactivity (CVR) were conducted using Transcranial Doppler (TCD Box, Compumedics DWL, Singen, Germany). We measured the blood flow velocity of the main intracranial arteries: the right and left middle cerebral arteries (Vmca), anterior cerebral arteries (Vaca), posterior cerebral arteries (Vpca) and the basilar artery (Vbas). Gosling`s pulsatility index (PI) of these 7 intracranial arteries was calculated according to the following formula: PI = (Vmax –Vmin)/Vmean. CVR was also measured by TCD using as trigger stimuli a voluntary apnoea, (the so-called breath-holding test). We calculated the percentage of increase of blood flow velocity in the right middle cerebral artery after a voluntary 25-seconds apnea, as previously described [16].

In order to assess the existence of a potential vascular remodelling, we measured the intima-media thickness (IMT) of common carotid arteries (MyLab 25 Gold, Esaote, Geneve, Italy) as previously reported [17] and also the distance between adventitial layers of both common carotid arteries. We estimated the endothelial function performing the post-ischemic brachial vasodilation test (also known as flow-mediated brachial vasodilation or brachial hyperaemic vasodilation test), expressed as percentage of dilation in 60 seconds [18] and serum levels of biomarkers related with inflammation and endothelial dysfunction: soluble vascular cell adhesion molecule-1 (sVCAM-1), tumor necrosis factor-α (TNF-α), high-sensitive CRP (hs-CRP) and interleukin-6 (IL-6). Plasma levels of sVCAM-1 (ng/mL), TNF-α and IL-6 (pg/mL) were determined using specific cytokine antibodies (Biosource Europe, S.A., Belgium), while high-sensitive C-reactive protein (hs-CRP) (range 0.05 – 170 mg/L) was measured using an immunoturbidimetric assay (Tina-quant® C-reactive protein (latex) high sensitive assay, Roche, USA).

### Statistical analysis

Data were analysed with SPSS for Windows (version 17.0). Continuous variables were presented as mean ± SD. Comparations between Fabry patients and controls were performed using the Mann–Whitney rank sum test for non-normally distributed data and the t-test for normally distributed variables. Values of p <0.05 were considered significant.

## Results

Demographic and baseline characteristics were similar in both groups. Age of participants was 37,5 ± 13,4 in FD patients and 37,7 ± 14,0 in control subjects (mean age ± SD). There were no relevant differences regarding classical vascular risk factors (arterial hypertension, diabetes, dyslipidemia) between groups: one subject of the control group had arterial hypertension, while four patients with FD versus two subjects of the control group suffered dyslipidemia. Of note, only one patient had advanced kidney disease, in whom the lowest α-Gal A activity was detected.

### Brain hemodynamics

No differences were observed between groups in the flow velocities of the brain arteries or in their PI, nor in the anterior group (middle and anterior cerebral arteries) neither in the posterior ones (posterior and basilar arteries) (Table 1).

**Table 1:** Parameters of cerebral vascular function (blood flow and cerebral vasomotor reactivity

**Parameter**		**Healthy volunteers (n = 17)**	**Patients with FD (n = 10)**	**p value**
*Right middle cerebral artery*				
	Mean blood flow velocity (cm/s)	56.07 ± 10.36	56.82 ± 15.53	0.9
	Maximum blood flow velocity (cm/s)	87.58 ± 16.48	86.64 ± 21.66	0.9
	Pulsatility index	0.84 ± 0.15	0.80 ± 0.18	0.57
	*Apnea Test (%)*	56.79 ± 15.68	47.25 ± 15.36	0.21
*Left middle cerebral artery*				
	Mean blood flow velocity (cm/s)	59.21 ± 14.15	58.15 ± 16.95	0.9
	Maximum blood flow velocity (cm/s)	94.30 ± 22.09	88.85 ± 26.64	0.6
	Pulsatility index	0.89 ± 0.1402	0.83 ± 0.2161	0.4
*Right anterior cerebral artery*				
	Mean blood flow velocity (cm/s)	47.90 ± 11.82	49.18 ± 13.00	0.8
	Maximum blood flow velocity (cm/s)	75.97 ± 16.73	74.53 ± 25.22	0.9
	Pulsatility index	0.89 ± 0.11	0.84 ± 0.21	0.4
*Left anterior cerebral artery*				
	Mean blood flow velocity (cm/s)	48.95 ± 11.69	47.29 ± 16.57	0.8
	Maximum blood flow velocity (cm/s)	76.66 ± 15.13	70.61 ± 25.79	0.5
	Pulsatility index	0.87 ± 0.15	0.89 ± 0.23	0.8
*Right posterior cerebral artery*				
	Mean blood flow velocity (cm/s)	40.93 ± 8.22	37.80 ± 11.11	0.4
	Maximum blood flow velocity (cm/s)	64.85 ± 13.19	53.55 ± 18.00	0.1
	Pulsatility index	0.8897 ± 0.23	0.7651 ± 0.20	0.2
*Left posterior cerebral artery*				
	Mean blood flow velocity (cm/s)	37.84 ± 5.12	37.60 ± 10.11	0.9
	Maximum blood flow velocity (cm/s)	59.41 ± 7.48	56.25 ± 17.09	0.5
	Pulsatility index	0.86 ± 0.13	0.83 ± 0.20	0.8
*Basilar artery*				
	Mean blood flow velocity (cm/s)	41.60 ± 8.14	45.38 ± 12.90	0.4
	Maximum blood flow velocity (cm/s)	65.35 ± 12.52	64.02 ± 25.69	0.8
	Pulsatility index	0.86 ± 0.13	0.78 ± 0.20	0.3

apnea test = breath-holding test. Data are means ± SD.

In the evaluation of cerebral vasomotor reactivity (apnea test), we only observed a tendency toward a lower vasomotor reactivity in patients with FD than in the control group (Table 1).

### Vascular remodelling

We did not find differences between groups in the thickness (IMT) of both common carotid arteries nor in the distance between adventitial layers of these arteries (Table 2). Interestingly, no atherosclerotic plaques were found in the carotid arteries in any of the studied subjects.

**Table 2:** Parameters of systemic endothelial function and vascular remodelling

**Parameter**	**Healthy volunteers (n = 17)**	**Patients with FD (n = 10)**	***p *** **value**
IMT right common carotid artery (mm)	0.57 ± 0.07	0.60 ± 0.19	0.51
Distance between advential layers of right common carotid artery (mm)	6.25 ± 1.55	6.50 ± 0.91	0.71
IMT left common carotid artery (mm)	0.54 ± 0.94	0.62 ± 0.21	0.16
Distance between advential layers of left common carotid artery (mm)	6.45 ± 0.78	6.66 ± 0.91	0.62
Mean diameter of brachial artery (mm)	3.64 ± 0.67	3.38 ± 0.70	0.36
Brachial hyperaemic vasodilation test (60”)	16.85 ± 10.59	14.10 ± 11.62	0.54
us-CRP (mg/L)	1.74 ± 1.51	2.92 ± 1.88	0.14
IL-6 (pg/mL)	7.48 ± 2.15	6.34 ± 1.42	0.06
sVCAM-1 (ng/mL)	*714.47 ± 178.22*	*899.50 ± 217.24*	*0.02*
TNF-α (pg/mL)	4.41 ± 1.17	5.41 ± 1.66	0.11

IMT, intima-media thickness. Data are means ± SD. p < 0.05 compared to control patient.

### Endothelial dysfunction

**Figure 1:**
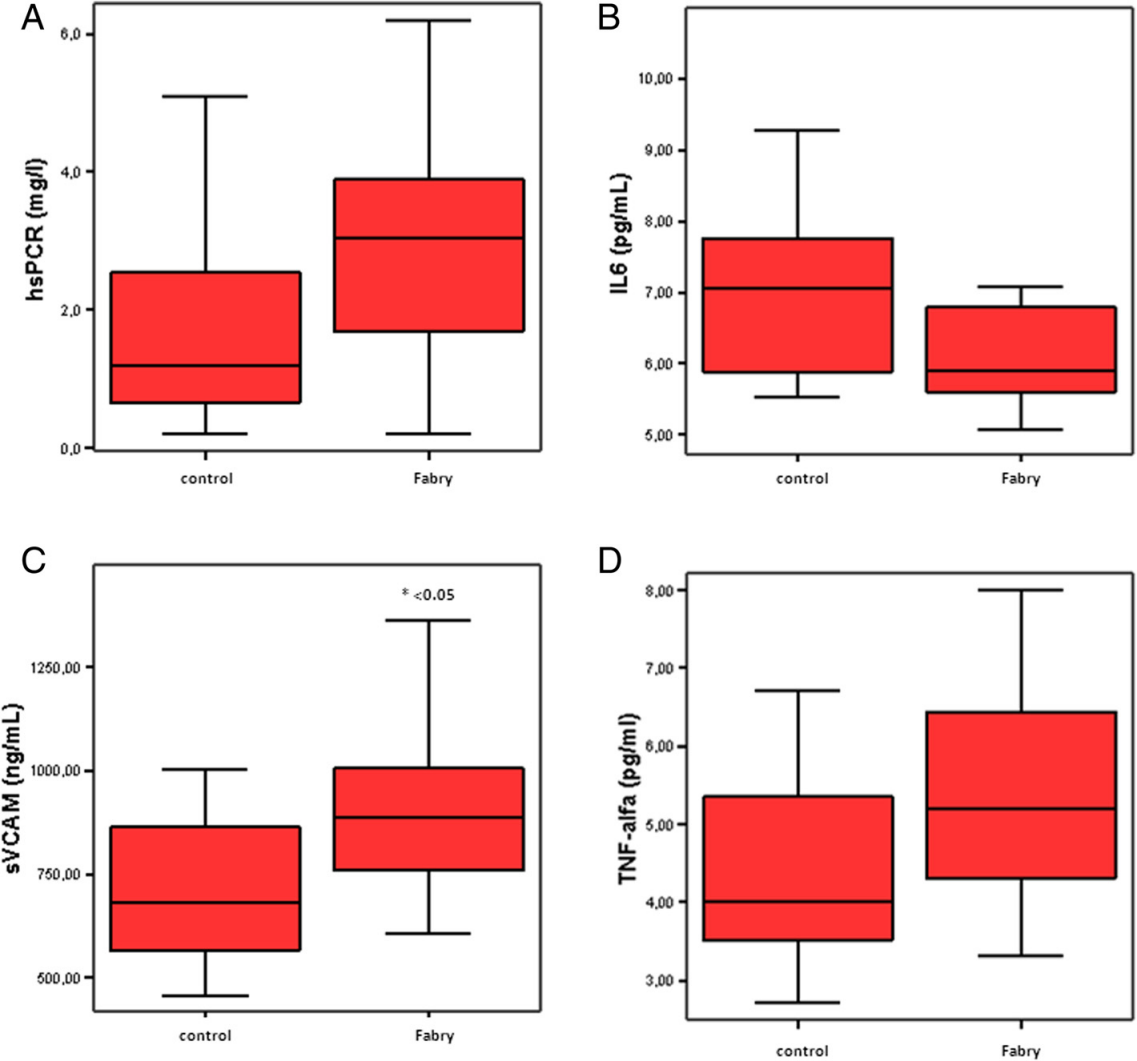
Charts showing mean values and 95% CIs of the plasma concentration levels of high-sensitive CRP (hs-CRP) (A), IL-6 (B), soluble VCAM (C) and TNF-alfa (D) in patients with Fabry disease (n = 10) and controls (n = 17), matched for age and gender. * p < 0.05.

In the study of post-ischemic brachial vasodilation, we did not find significant differences between groups in the percentage of dilation after 60 seconds (Table 2). However, concerning serum levels of biomarkers for endothelial dysfunction and inflammation, we found low serum levels of IL-6 and high serum levels of sVCAM-1, TNF-α and hs-CRP in Fabry patients than in healthy volunteers. Notably, in the case of sVCAM-1, statistically significant differences were observed (899.50 ng/mL in FD patients vs 714.47 ng/mL in healthy volunteers, p = 0.02) (Table 2 and Figure 1).

## Discussion

The main clinical manifestations in FD consist of vascular associated complications, but the pathophysiology of the FD vasculopathy is unclear. Specifically, the exact nature of the cerebrovascular disease in FD is controversial. It has been postulated that the accumulation of glycosphyngolipids in the endothelium might increase the risk of stroke directly by provoking thickening with occlusion of the small vessels or indirectly by generating endothelial dysfunction [14]. However, there is also the possibility that the anomalous accumulation of glycosphyngolipids may affect the muscle cells of the vessel wall, and/or the vegetative neurons regulating them [19]. If this latter occurred, there would be a loss of the characteristic special hemodynamics of the brain vessels, including vasoreactivity [12,14,20]. Although it would be expected that this disturbance only was able to affect the smaller vessels of the brain, the fact is that previously some authors had described the existence of hyperflow [14,21] in the basal cerebral arteries, while others found hypoflow [13]. In our study we have not found relevant differences in the blood flow parameters of the main cerebral arteries between healthy volunteers and FD patients. These findings may be related to the small sample size, although the results did not show any trend. Secondly, we studied if FD patients suffered any alteration of the cerebral vasoreactivity. We only detect a trend toward a lower vasomotor reactivity in the middle cerebral artery of patients with FD. The cerebral vasomotor reactivity is a control mechanism to regulate the brain blood supply in an attempt to ensure that the metabolism of the brain will not be affected by systemic alterations or arterial pressure variations. It achieves its function by increasing the blood flow through vasodilation of the cerebral arterioles if the systemic pressure falls or the metabolic conditions worsen. In fact, cerebrovascular reactivity damage due to autonomic dysfunction has been previously considered among the pathogenic factors of thrombotic and ischemic brain infarctions of FD [12,13], but in our group, we were not able to confirm that hypothesis. Again, these findings may be related to the small sample size or be influenced because the measures were taken at the middle cerebral artery. Thus, a recent study [15], did find a disturbed neurovascular coupling in the territory of the posterior cerebral artery, which could translate a dysfunction of cerebrovascular reactivity in the territory of that artery.

Our work was also designed to investigate whether arterial remodelling could influence the high vascular risk of Fabry patients. As such, we measured the inter-adventicial caliber of the carotid arteries and the thickness of the intima-media layer. We found no differences between groups. It therefore seems unlikely that macrovascular damage in the extracranial bed could be responsible for the origin of cerebrovascular disease in most Fabry patients, at least until advanced stages of disease.

Finally, we also investigated the presence of endothelial dysfunction. First, we used a test of proven value in determining endothelial dysfunction, the brachial hyperaemic vasodilation test. We did not find relevant differences between patients and controls. Previous studies suggested a chronic alteration of the nitric oxide (NO) pathway in Fabry disease [14], but at the same time has been shown that non-NO factors determine an enhanced endothelium-dependent vasodilation in Fabry disease [22]. Thus, an event could compensate for another and the final result may be a normal brachial hyperaemic vasodilation test, as we found. Secondly, serological markers of inflammation and endothelial activation were measured. Probably the most relevant finding of our study was that, in absence of clear vascular remodelling or vasodilatadory endothelial dysfunction, increased levels of endothelium-release biomarkers (sVCAM-1, hs-CRP and TNF-α) were observed in patients with FD. In the case of sVCAM-1, the differences were statistically relevant. Previous studies had also reported increased biomarker levels for endothelial activation, such as sVCAM-1, sICAM, P-selectin, E-selectin, plasminogen activator inhibitor (PAI) and endothelium derived micro-particles [23-26], thus suggesting that a local process in the vessel wall could be primarily responsible for the ischemic lesions [19]. In an in vitro study [25], an excess of intracellular accumulation of Gb3 induced oxidative stress and up-regulated the expression of cellular adhesion molecules (ICAM-1, VCAM-1 and E-Selectin) in vascular endothelial cells of FD patients. Reduction of endogenous Gb3 by treatment of the cells with an inhibitor of glycosphingolipid synthase or α-galactosidase A led to decreased expression of adhesion molecules. At a clinical level, a study done investigating 25 patients and 25 controls [23] showed increased concentrations of sICAM-1, sVCAM-1, P-selectin and PAI, and decreased levels of thrombomodulin in Fabry patients, thus suggesting that endothelium activation and a prothrombotic state may play a significant role in the occurrence of stroke in Fabry disease. However, in a subsequent study [24] no differences were found in ICAM-1, plasma P-selectin or plasma thrombomodulin levels between 12 patients and 15 controls, although plasmatic levels of sVCAM-1 were also significantly higher in patients with Fabry disease.

We also found higher levels of sVCAM-1, hs-CRP and TNF-α in FD patients than in healthy volunteers and in the case of sVCAM-1, the differences were statistically relevant. The VCAM-1 molecule is inducible by cytokines such as TNF-α, IL-1, and IL-4 on endothelium in vitro [27]. VCAM-1 mediates the adhesion of immflamatory cells to vascular endothelium and functions in leukocyte-endothelial cell signal transduction [28]. For these reasons, VCAM-1 is a valid candidate marker to measure endothelial dysfunction in vivo, but its high levels should be considered as an indicator of systemic endothelial dysfunction and not just a local phenomenon [19,29].

Our study has some limitations. First, the small size of our sample. FD is a rare condition and it is difficult to get a large number of patients to study (*in Spain, for example, little more than 100 patients have been diagnosed)*. Then, a complete and centralized vascular study over 10 patients should be considered of interest. Another possible limitation of this study is the pooled analysis of data from both homozygous and hemizygous FD patients and from patients receiving ERT and untreated patients. Nevertheless, in subanalyses performed only in homozygous or hemizygous alone or in treated/untreated patients, similar results were obtained (data not showed). Finally, it has been proposed that the plasmatic abnormalities in markers for endothelial activation were more likely related to renal insufficiency rather than to Fabry disease itself [30]. However, in our sample only one patient had advanced kidney disease, and both the remaining patients and controls had a glomerular filtration rate within normal limits. Excluding this sole patient in the statistical analysis did not change the differences between the groups regarding to levels of sVCAM-1. In fact, the detected value of sVCAM-1 in this patient was 662 ng/mL, below the mean value of patients and controls.

Our findings support the hypothesis that an increased endothelial inflammatory profile exists in Fabry disease patients. This proinflammatory endothelial dysfunction seems to be present in the early stages of the illness, before any sign of atheromatosis or arterial remodelling and even before any relevant alteration of the endothelium-dependent vasodilator capacity, and could be the main cause of the thrombogenicity of FD. If so, then serum biomarkers are probably better indicators than brain hemodynamic parameters to estimate the stroke risk in FD patients and to measure the efficacy of therapeutic interventions in this field.

Our results do not help to explain the special affinity of FD thrombogenicity by the brain. We cannot exclude that in patients with advanced disease a clear deterioration of cerebral hemodynamics could appear; that situation, coupled with the previous endothelial dysfunction might then explain the predominant involvement of the brain. To clarify this hypothesis, larger studies specially focused in FD patients who have already had cerebral involvement, are necessary.

## Conclusions

In summary, the vasculopathy of Fabry disease has been classically attributed to the progressive deposition of Gb3 in the vascular endothelium, which could eventually lead to structural abnormalities in the vessel wall; however, our results as well as other from previous studies, allow us to hypothesize that the primary metabolic defect could induce a cascade of events leading to endothelial dysfunction that, with the progression of the disease, might promote thrombotic phenomena and cerebrovascular events. Serum biomarkers would then be probably better indicators than brain hemodynamic parameters to estimate the stroke risk in Fabry disease and to measure the efficacy of therapeutic interventions in this field.

## Abbreviations

sVCAM-1: Soluble vascular cell adhesion molecule-1; TNF-α: Tumor necrosis factor-α; hs-CRP: High-sensitive C-reactive protein; IL-6: Interleukin-6; FD: Anderson Fabry disease; GLA: Alpha-galactosidase A; Gb3: Globotriaosylceramide; TIA: Transient ischemic attack; ERT: Enzyme replacement therapy; TCD: Transcranial doppler; FOS: Fabry outcome survey; CVR: Cerebral vasomotor reactivity; Vmca: Mean velocity of middle cerebral artery; Vaca: Mean velocity of anterior cerebral artery; Vpca: Mean velocity of posterior cerebral artery; Vbas: Mean velocity of basilar cerebral artery; PI: Pulsatility index; IMT: Intima-media thickness; NO: Nitric oxide; sICAM: Soluble intercellular adhesion molecule; PAI: Plasminogen activator inhibitor; IL-1: Interleukin-1; IL-4: Interleukin-4.

## Competing interests

Dr Segura has served as a consultant for Shire Human Genetic Therapies, Inc. The other authors report no conflicts.

## Authors’ contributions

TS selected the patients, evaluated their clinical data, performed the statistical analysis, prepared the manuscript draft and participated in the study design. OAM analyzed the ultrasound data, participated in the study design and helped drafting the manuscript. IGF verified and evaluated the clinical data of the patients and participated in creating the database. CA helped in the selection of patients and controls and analyzed the laboratory data. MAB helped in the selection of patients and participated in the study design. JV analyzed the ultrasound data and participated in the coordination of the study. All authors read and approved the final manuscript.

## Pre-publication history

The pre-publication history for this paper can be accessed here:

http://www.biomedcentral.com/1471-2377/13/170/prepub

## References

[B1] Burlina AP Sims KB Politei JM Bennett GJ Baron R Sommer C Early diagnosis of peripheral nervous system involvement in Fabry disease and treatment of neuropathic pain: the report of an expert panel BMC Neurol 2011 11 61 10.1186/1471-2377-11-61 21619592 PMC3126707

[B2] Celtikci B Topçu M Ozkara HA Two novel alpha-galactosidase a mutations causing fabry disease: a missense mutation M11V in a heterozygote woman and a nonsense mutation R190X in a hemizygote man Clin Biochem 2011 44 809 812 10.1016/j.clinbiochem.2011.04.022 21569769

[B3] Lidove O Kaminsky P Hachulla E Leguy-Seguin V Lavigne C Marie I Fabry disease 'The New great Imposter’: results of the French observatoire in internal medicine departments (FIMeD) Clin Genet 2012 81 571 577 10.1111/j.1399-0004.2011.01718.x 21623772

[B4] Altarescu G Elstein D Fabry disease in an oligosymptomatic male Isr Med Assoc J 2011 13 191 192 21608346

[B5] Ramaswami U Update on role of agalsidase alfa in management of Fabry disease Drug Des Devel Ther 2011 5 155 173 21552486 10.2147/DDDT.S11985 PMC3084298

[B6] Brouns R Thijs V Eyskens F Van den Broeck M Belachew S Van Broeckhoven C Belgian Fabry study: prevalence of Fabry disease in a cohort of 1000 young patients with cerebrovascular disease Stroke 2010 41 863 868 10.1161/STROKEAHA.110.579409 20360539

[B7] Alfadhel M Sirrs S Enzyme replacement therapy for Fabry disease: some answers but more questions Ther Clin Risk Manag 2011 7 69 82 21445281 10.2147/TCRM.S11987 PMC3061846

[B8] Salviati A Burlina AP Borsini W Nervous system and Fabry disease, from symptoms to diagnosis: damage evaluation and follow-up in adult patients, enzyme replacement, and support therapy Neurol Sci 2010 31 299 306 10.1007/s10072-009-0211-y 20300794 PMC2869001

[B9] Aerts JM Kallemeijn WW Wegdam W Joao Ferraz M van Breemen MJ Dekker N Biomarkers in the diagnosis of lysosomal storage disorders: proteins, lipids, and inhibodies J Inherit Metab Dis 2011 34 605 619 10.1007/s10545-011-9308-6 21445610 PMC3109260

[B10] Rolfs A Böttcher T Zschiesche M Morris P Winchester B Bauer P Prevalence of Fabry disease in patients with cryptogenic stroke: a prospective study Lancet 2005 366 1794 1796 10.1016/S0140-6736(05)67635-0 16298216

[B11] MacDermot KD Holmes A Miners AH Anderson-Fabry disease: clinical manifestations and impact of disease in a cohort of 60 obligate carrier females J Med Genet 2001 38 769 775 10.1136/jmg.38.11.769 11732485 PMC1734754

[B12] Kolodny EH Pastores GM Anderson-Fabry disease: extrarenal, neurologic manifestations J Am Soc Nephrol 2002 13 Suppl 2 S150 S153 12068029

[B13] Hilz MJ Kolodny EH Brys M Stemper B Haendl T Marthol H Reduced cerebral blood flow velocity and impaired cerebral autoregulation in patients with Fabry disease J Neurol 2004 251 564 570 10.1007/s00415-004-0364-9 15164189

[B14] Moore DF Scott LT Gladwin MT Altarescu G Kaneski C Suzuki K Regional cerebral hyperperfusion and nitric oxide pathway dysregulation in Fabry disease: reversal by enzyme replacement therapy Circulation 2001 104 1506 1512 10.1161/hc3801.096352 11571244

[B15] Azevedo E Mendes A Seixas D Santos R Castro P Ayres-Basto M Rosengarten B Oliveira JP Functional transcranial Doppler: presymptomatic changes in Fabry disease Eur Neurol 2012 67 331 337 10.1159/000337906 22572628

[B16] Jiménez-Caballero PE Segura T Normal values of cerebral vasomotor reactivity using the breath-holding test Rev Neurol 2006 43 598 602 17099851

[B17] Touboul PJ Hennerici MG Meairs S Adams H Amarenco P Bornstein N Csiba L Desvarieux M Ebrahim S Hernandez Hernandez R Jaff M Kownator S Naqvi T Prati P Rundek T Sitzer M Schminke U Tardif JC Taylor A Vicaut E Woo KS Mannheim carotid intima-media thickness and plaque consensus (2004-2006-2011). an update on behalf of the advisory board of the 3rd, 4th and 5th watching the risk symposia, at the 13th, 15th and 20th European stroke conferences, Mannheim, Germany, 2004, Brussels, Belgium, 2006, and Hamburg, Germany, 2011 Cerebrovasc Dis 2012 34 290 296 10.1159/000343145 23128470 PMC3760791

[B18] Corretti MC Anderson TJ Benjamin EJ Celermajer D Charbonneau F Creager MA Guidelines for the ultrasound assessment of endothelial-dependent flow-mediated vasodilation of the brachial artery: a report of the international brachial artery reactivity task force J Am Coll Cardiol 2002 39 257 265 11788217 10.1016/s0735-1097(01)01746-6

[B19] Rombach SM Twickler TB Aerts JM Linthorst GE Wijburg FA Hollak CE Vasculopathy in patients with Fabry disease: current controversies and research directions Mol Genet Metab 2010 99 99 108 10.1016/j.ymgme.2009.10.004 19900828

[B20] Itoh Y Esaki T Cook M Qasba P Shimoji K Alroy J Local and global cerebral blood flow and glucose utilization in the alpha-galactosidase a knockout mouse model of fabry disease J Neurochem 2001 79 1217 1224 11752062 10.1046/j.1471-4159.2001.00669.x

[B21] Politei JM Capizzano AA Magnetic resonance image findings in 5 young patients with Fabry disease Neurologist 2006 12 103 105 10.1097/01.nrl.0000187495.16824.a6 16534447

[B22] Altarescu G Moore DF Pursley R Campia U Goldstein S Bryant M Enhanced endothelium-dependent vasodilation in Fabry disease Stroke 2001 32 1559 1562 10.1161/01.STR.32.7.1559 11441201 PMC4770460

[B23] DeGraba T Azhar S Dignat-George F Brown E Boutière B Altarescu G Profile of endothelial and leukocyte activation in Fabry patients Ann Neurol 2000 47 229 233 10.1002/1531-8249(200002)47:2<229::AID-ANA13>3.0.CO;2-T 10665494

[B24] Demuth K Germain DP Endothelial markers and homocysteine in patients with classic Fabry disease Acta Paediatr Suppl 2002 91 57 61 12572844 10.1111/j.1651-2227.2002.tb03112.x

[B25] Shen JS Meng XL Moore DF Quirk JM Shayman JA Schiffmann R Globotriaosylceramide induces oxidative stress and up-regulates cell adhesion molecule expression in Fabry disease endothelial cells Mol Genet Metab 2008 95 163 168 10.1016/j.ymgme.2008.06.016 18707907 PMC2593623

[B26] Gelderman MP Schiffmann R Simak J Elevated endothelial microparticles in Fabry children decreased after enzyme replacement therapy Arterioscler Thromb Vasc Biol 2007 27 e138 e139 10.1161/ATVBAHA.107.143511 17581828

[B27] Osborn L Leukocyte adhesion to endothelium in inflammation Cell 1990 62 3 6 10.1016/0092-8674(90)90230-C 2194672

[B28] Tokuhira M Hosaka S Volin MV Haines GK Katschke KJ Kim S Soluble vascular cell adhesion molecule 1 mediation of monocyte chemotaxis in rheumatoid arthritis Arthritis & Rheumatism 2000 43 1122 1133 10.1002/1529-0131(200005)43:5<1122::AID-ANR23>3.0.CO;2-7 10817567

[B29] Constans J Conri C Circulating markers of endothelial function in cardiovascular disease Clin Chim Acta 2006 368 33 47 10.1016/j.cca.2005.12.030 16530177

[B30] Vedder AC Biró E Aerts JM Nieuwland R Sturk G Hollak CE Plasma markers of coagulation and endothelial activation in Fabry disease: impact of renal impairment Nephrol Dial Transplant 2009 24 3074 3081 10.1093/ndt/gfp263 19515805

